# Kinetics and Optimization of Metal Leaching from Heat-Resistant Nickel Alloy Solid Wastes

**DOI:** 10.3390/molecules28145545

**Published:** 2023-07-20

**Authors:** Imran Ali, Anastasya Gaydukova, Tatiana Kon’kova, Zeid Abdullah ALOthman, Mika Sillanpää

**Affiliations:** 1 Department of Chemistry, Jamia Millia Islamia (Central University), Jamia Nagar, New Delhi 110025, India; 2 Department of Inorganic Substances Technology and Electrochemical Processes, Mendeleev University of Chemical Technology, 9 Miusskaya Sq., Moscow 125047, Russia; 3 Department of Chemistry, College of Science, King Saud University, Riyadh 11451, Saudi Arabia; zaothman@ksu.edu.sa; 4 Department of Biological and Chemical Engineering, Aarhus University, Norrebrogade 44, 8000 Aarhus C, Denmark

**Keywords:** recoveries of metals, heat-resistant nickel alloys waste, acid leaching, kinetics of leaching, optimization of the leaching process

## Abstract

Recycling waste from the production and consumption of heat-resistant alloys to return them to production is an urgent task due to the high cost of the components contained in these alloys. The kinetics and conditions of the acid leaching process of the grinding waste of a heat-resistant nickel alloy are studied depending on the composition of the acid solution (H_2_SO_4_, HCl, HNO_3_, and their mixtures) at room temperature to boiling point temperature and various acid concentrations (1.5 to 3.0 mol/L), ratios of waste to solution (1:10 to 3:10), fraction sizes (0.04–1 mm), and contact duration (1 to 120 h). The linearization of experimental data by the Gray–Weddington, Gistling–Brownstein, and Kazeev–Erofeev equations showed that the rate of the leaching process was influenced by both the chemical reactions between sulfuric acid and metals included in the grinding waste and the diffusion of reagents through the film of reaction products and undissolved impurities. Optimal conditions for acid dissolution of the grinding waste have been established to obtain the maximum degree of extraction of the main component of the alloy, nickel. The processing of powder particles with a size of less than 0.1 mm should be carried out in a solution of sulfuric acid with a concentration of 3.0 mol/L at a temperature of 100 °C for 6 h with a ratio of solid to liquid phases of 1:10. The reported results are very important for industry personnel to recover metals and for environmentalists to treat the alloy waste.

## 1. Introduction

The presence of toxic metal ions in any environment is unacceptable due to their acute toxicity [1,2,3]. Every year, as a result of the cutting and grinding of hard alloy products, industrial companies produce a significant amount of waste, which is finely dispersed waste material. In addition, to date, a large number of hard alloy products with expired service lives have accumulated around the world. Heat-resistant nickel alloys belong to hard alloys and occupy a leading place among other materials used in machines, aircraft, and other industries. The direct processing of products made of heat-resistant alloys that have fallen out of use, as well as grinding waste from their production, is an urgent task since it is due to the high cost of the components contained in these alloys and the need to return them to production [4]. Along with cobalt, molybdenum, and aluminum, the heat-resistant nickel alloy contains other valuable and refractory metals, such as tungsten, niobium, and rhenium. The approximate composition of nickel alloy (wt.%) is as follows: Cr, 4.9–5.0; Co, 9–9.3; Mo, 1–1.1; W, 8.5; Al, 5.9–6.0; Ta, 4.0; Nb, 1.6; Re, 4.0; Ni, 57.4–60 [5,6].

The share of valuable metals in grinding waste is significantly less than in products with a hard surface due to their heavy contamination with cutting lubricants, worn grinding media, and grinding products made of iron-based alloys. The proportion of impurities varies widely; for example, the amount of abrasive in the waste can be from 4 to 20%, lubricants (oil) from 5 to 40%, and water up to 30% [7]. There are three areas of processing of metal-containing waste and grinding waste of hard alloys: pyrometallurgical, hydrometallurgical, and combined. Pyrometallurgical treatment is carried out both by traditional methods at atmospheric pressure at temperatures of 800–2000 °C and using vacuuming (10^−2^ mbar) or increased pressure up to 100 MPa. In the latter case, the process is carried out in autoclaves [7]. Hydrometallurgical methods, in turn, are divided into chemical and electrochemical. Scientific research presents the results of biological leaching of valuable metals from man-made waste, but this method has not found wide industrial applications [8]. The electrochemical method is more often used for the decomposition of scrap or large-scale waste, which, with the correct choice of design of the electrolyzer and electrolyte, allows metals to be transferred to a solution with sufficiently high technological parameters [9]. For powder-type waste, it is advisable to use reagent dissolution, since the small size of their fractions can have a positive effect on the kinetics of the transition to the solution of the main component of the grinding waste—nickel.

Along with traditional reagents for hydrometallurgical leaching of metals, such as mineral acids (nitric, sulfuric, and hydrochloric) [10,11,12,13], in scientific research, it is proposed to use deep eutectic solvents for this purpose, e.g., choline chloride, carbamide, and carboxylic acids (citric and lactic), having proton acceptor or proton-donating ability [14], and ligand-forming reagents (EDTA) [15]. However, the efficiency of the latter is low compared to mineral acids, and it is unlikely that they will find real practical application. To remove organic impurities and increase the efficiency of the leaching process, heat treatment of the material is carried out, for example, alkaline melting using sodium carbonate [16]. The advantage of the pyrometallurgical method is the absence of wastewater, but the disadvantage is the lack of selectivity in obtaining a commercial product. Hydrometallurgy is more selective and can provide almost complete separation of elements by methods of leaching, crystallization, liquid extraction, adsorption on inorganic materials of synthetic and natural origin, and ion exchange resins [17,18,19,20,21,22,23]. However, there are a number of difficulties associated, for example, with the separation of liquid and solid, the selective extraction of elements with similar properties from the leaching solution (cobalt and nickel), and the high costs of reagents [24,25].

There are two approaches to the processing of metal-containing waste. One of them consists of the step-by-step extraction of alloy components into an electrolyte solution, followed by their separation from the solution. The other approach involves the joint transfer of all valuable alloy components into solution and their subsequent selective isolation in the form of poorly soluble compounds, for example, when adjusting the pH of solutions with subsequent filtration or selective separation by the above methods [26,27]. The second approach is the simplest in technological execution. An analysis of the literature devoted to the processing of the waste of heat-resistant nickel alloys has shown that acid dissection is the main problem of the hydrometallurgical processing process. Nickel is soluble in mineral acid solutions only under certain conditions. The rate of its transition to a solution, regardless of the method of dissolution (chemical or electrochemical), increases with an increase in the temperature of the process and the concentration of acids to certain values at which its passivation is observed [6]. Due to the heterogeneity and differences in the chemical composition of waste heat-resistant alloys, passivation of the alloy surface during leaching and slowing down the process speed, as well as the not always possible adaptation of the literature data, the choice of methods, conditions for their opening, and further separation of the main components are determined experimentally for a specific waste. The purpose and novelty of the work consist of working out the mode of chemical leaching of the main component of nickel from the waste of a heat-resistant nickel alloy in order to isolate it as completely as possible and selecting optimal specific process parameters.

## 2. Results and Discussion

### 2.1. Optimization of the Acid Decomposition Process of Grinding Waste

The granulometric compositions of the particles of the initial and heat-treated grinding wastes according to the sieve analysis were determined. The size distribution of the grinding waste particles based on the sieve analysis is presented in Table 1. The powder in its bulk consists of two fractions in the range of 0.2–0.5 and 0.1–0.2 mm. There is also a small number of particles with sizes of 0.063–0.1 mm and 0.5–1 mm.

**Figure 1:**
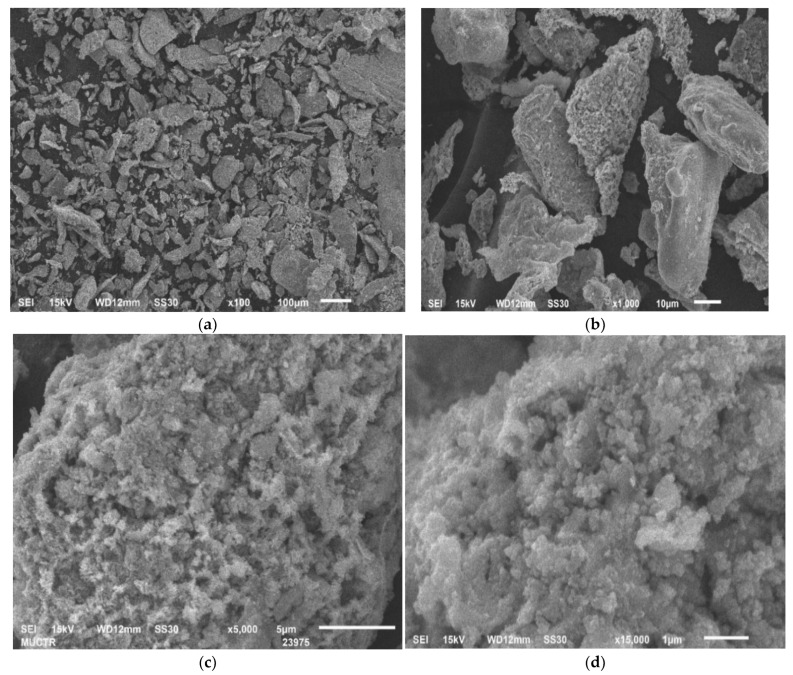
Micrographs of the initial grinding waste powder at magnification: (**a**) 100 times; (**b**) 1000 times; (**c**) 5000 times; (**d**) 15,000 times.

The morphology and dispersed composition of the grinding waste particles obtained by scanning electron microscopy at different magnifications are shown in Figure 1. It can be seen from the obtained microphotographs that the powder under study was heterogeneous in its dispersed and morphological compositions and contained mainly irregularly shaped particles with a loose surface and a size in the range of 10–100 microns.

According to the results of X-ray energy dispersion spectroscopy (Table 2) in the initial sample, the main element was nickel. Also, chromium, tungsten, and molybdenum were present. In addition, aluminum, titanium, and cobalt were also found in insignificant amounts. The presence of carbon indicated the occurrence of carbides of these metals and organic impurities. The presence of oxygen was due to the presence of oxidized forms of metals.

For a more complete analysis of the powder by the content of metals, especially those present in microquantities, the method of inductively coupled plasma optical emission spectroscopy was used. For this, the sample was dissolved in hydrochloric acid when exposed to microwave radiation. From the data obtained (Table 3), it can be seen that in addition to the above basic elements, valuable elements were also present in the powder, i.e., rhenium, iridium, vanadium, and ruthenium. It was also noted that the high content of rhenium in the grinding waste was 5.9 g/kg. Both methods of elemental analysis used in this case were evaluative and complementary since the first method allowed us to determine the elemental composition of individual sections of the sample surface. In the second, there was an incomplete dissolution of the powder, resulting in a certain amount of undissolved residue. Therefore, in general, the results are somewhat different, especially in the content of trace impurities. In addition, the uneven distribution of impurities over the volume of the alloy also led to a difference in the results.

Since the grinding waste always contains lubricating and cooling fluids, which is confirmed by the results of elemental analysis, according to which the carbon content was almost 15 wt.% (Table 2), their presence made it difficult for the reagent to access the components to be opened. In addition, the transition of organic impurities into the solution will eventually significantly worsen the quality of the final product. Indeed, after opening the waste with a solution of sulfuric acid at a temperature of 100 °C, the COD index indicating the content of organic carbon in the solution was 40,000 mg/L. To remove organic impurities, the initial powder was subjected to heat treatment at 600 °C for 2 h. As a result, during its subsequent acid treatment under the same conditions, the content of organic carbon in the solution decreased three times and amounted to 12,000 mg/L.

**Figure 2:**
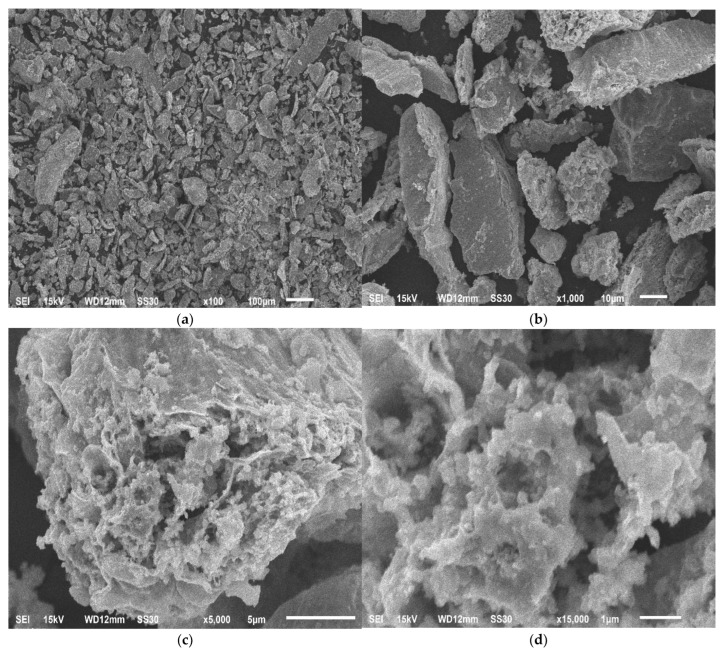
Micrographs of heat-treated grinding waste powder at magnification: (**a**) 100 times; (**b**) 1000 times; (**c**) 5000 times; (**d**) 15,000 times.

Repeated sieve analysis of heat-treated grinding waste showed (Table 1) that this operation contributes to an increase in the proportion of particles of a smaller fraction; a fraction with a particle size of less than 0.05 mm was also formed, the proportion of which was 27.36 wt.%. Scanning electron microscopy micrographs of the material (Figure 2c,d) indicate that after heat treatment of the powder, as a result of the removal of organic impurities, the particle structure became looser. According to X-ray energy dispersion spectroscopy data, as a result of heat treatment of the grinding waste sample, it is possible to remove most of the organic impurities, while the carbon content was significantly reduced (Table 2).

**Figure 3:**
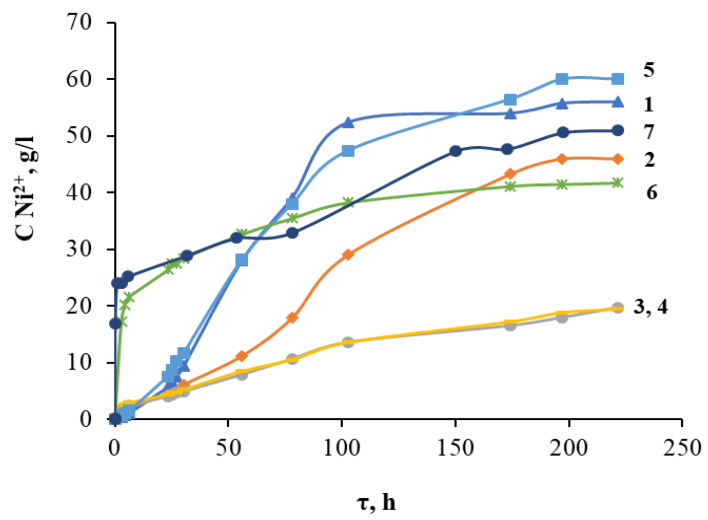
Dependence of the concentration of nickel ions in solution on the duration of acid treatment of the powder and the composition of the medium at room temperature (T = 25 °C): 1—3.0 mol/L H_2_SO_4_; 2—6.0 mol/L HCl; 3—6.0 mol/L HNO_3_; 4—3.0 mol/L H_2_SO_4_ + 0.3 mol/L HNO_3_; 5—3.0 mol/L H_2_SO_4_ +0.3 mol/L HCl; 6—6.0 mol/L HNO_3_ + 0.3 mol/L HCl; 7—6.0 mol/L HCl + 0.3 mol/L HNO_3_.

To study the process of chemical dissolution of the grinding waste powder, seven solutions were selected, including sulfuric, hydrochloric, and nitric acids and their mixtures. Due to the fact that the basis of the grinding waste was nickel, the control of its dissolution was analyzed by the concentration of nickel (II) ions in the solution. The data obtained by dissolving the powder at room temperature are shown in Figure 3.

It was found that in solutions containing a mixture of nitric and hydrochloric acids (curves 6 and 7), in the first hours of processing, the speed of the powder dissolution process was maximal due to the formation of the active form of HNO_2_. After 100 h, the dissolution rate slowed down and the kinetic curve reached a plateau, while the concentration of nickel in a solution of 6.0 mol/L HNO_3_ + 0.3 mol/L HCl did not exceed 42 g/L (at the same time Ni leaching efficiency of 65.6%), and in a solution of 6.0 mol/L HCl + 0.3 mol/L HNO_3_ − 51 g/L (Ni leaching efficiency of 79.7%). The slowing of the processing speed was due to the passivation of the powder surface by an oxide film. In solutions containing nitric acid, as well as a mixture of sulfuric and nitric acid, the dissolution process was the slowest and generally the least effective, as a result of which the nickel concentration in the solution did not exceed 20 g/L (Ni leaching efficiency of 1.3%) even after 220 h of treatment. The maximum concentration of nickel ions in the solution was observed after processing the metal powder in a solution containing a mixture of 3.0 mol/L H_2_SO_4_ and 0.3 mol/L HCl and was 60 g/L (Ni leaching efficiency of 93.8%). A slightly lower nickel ion content of 56 g/L (leaching efficiency of 87.5%) was obtained in a solution of 3.0 mol/L H_2_SO_4_. To reduce the corrosive activity of the solution due to the presence of hydrochloric acid, further studies were carried out using sulfuric acid.

**Figure 4:**
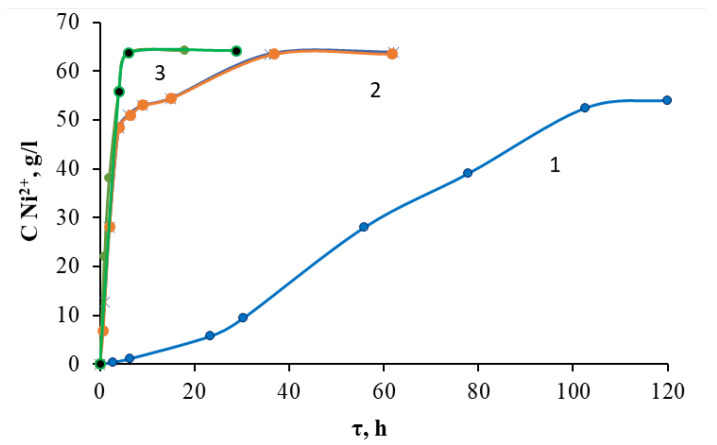
Kinetic curves of nickel ions leaching into solution in H_2_SO_4_ medium with a concentration of 3.0 mol/L depending on the temperature of the solution: 1—25 °C; 2—70 °C; 3—100 °C.

The temperature of the process is an important parameter for increasing the efficiency of dissolution. On the one hand, its increase contributes positively to the increase in the degree of extraction. On the other hand, its high value can lead to increased energy consumption. Experiments to find the optimal temperature of the process were carried out in a solution of sulfuric acid with a concentration of 3 mol/L. The efficiency of the process was evaluated by the extraction of nickel from the solution, and the results are shown in Figure 4.

From the data obtained, it can be seen that when solutions were heated from room temperature to 70 °C for the first 6 h, the nickel concentration in the solution increased from 2–3 g/L to 30–50 g/L (at the same time, Ni leaching efficiency was 46.9–78.1%). An increase in temperature to 100 °C increased the concentration of nickel ions in solution to 56 g/L in the first 4 h from the start of dissolution; in 6 h of contact, the concentration of nickel ions reaches 64 g/L. This is almost 100% leaching efficiency. At a temperature of 70 °C, a similar leaching efficiency was achieved in a time interval of more than 36 h. Thus, in terms of time and energy costs, the process should be carried out at boiling point. The implementation of autoclave leaching at 130 °C reduced the process time to 2 h while maintaining the efficiency of nickel leaching at almost 100%. Despite the fact that the autoclave process is more complicated in hardware design, it is probably the autoclave process that should be used in industrial conditions.

Along with the main component of the nickel alloy, other components and impurities are extracted into the solution during leaching, to varying degrees due to their solubility under these conditions (Table 4).

For example, chromium, cobalt, aluminum, titanium, and iron were leached very efficiently, while rhenium practically remained undissolved. It can be noted that with comparable amounts of iron and rhenium in the initial powder, when leached with sulfuric acid, rhenium passed into the solution at a concentration 20 times lower than iron. According to the literature data [6], indeed, as a result of the treatment of the alloy with sulfuric acid at a temperature of 85 °C, the leaching of rhenium into the solution did not exceed 5%. For effective leaching of rhenium, it is necessary to carry out electrochemical dissolution of the grinding waste, while the efficiency of rhenium extraction into solution reaches about 80% [28], or use oxidants such as chlorine, in which case the degree of rhenium extraction increases to 90% [29]. However, in our case, the preservation of vision in the undissolved residue allows you to concentrate it, separating it from the soluble elements, thereby making it possible to carry out its subsequent processing. The concentration of molybdenum in the solution was extremely low. Which is also consistent with literary sources [9]. Vanadium, iridium, ruthenium, and calcium were not detected in the solution, respectively. They remained in the undissolved residue, while niobium, tantalum, and sodium appeared in the solution.

**Figure 5:**
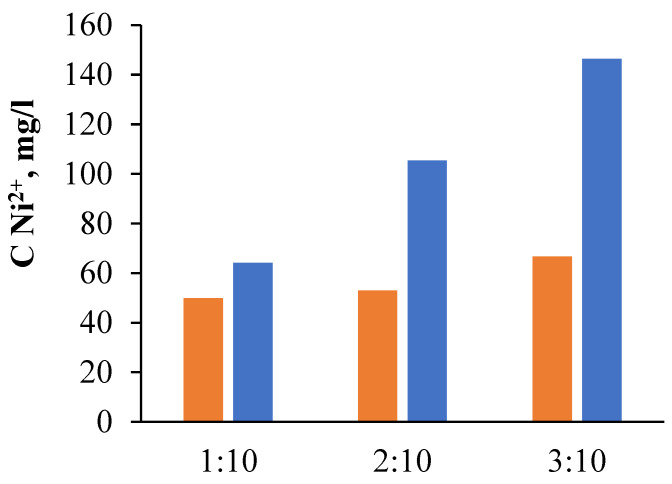
Dependence of nickel ion concentration in solution on the ratio of waste mass to solution volume after 10 h of treatment in H_2_SO_4_ solution at a temperature of 100 °C: orange color—acid concentration 1.5 mol/L; blue color—acid concentration 3.0 mol/L.

As a result of the studies conducted on the effect of acid concentration on the efficiency of metal leaching from the grinding waste, it was established (Figure 5, Table 5) that at a concentration of sulfuric acid of 1.5 mol/L, the concentration of nickel ions in solution after 10 h at a temperature of 100 °C was 50 g/L, while with an increase in the concentration of sulfuric acid to 3.0 mol/L, the concentration of nickel ions increased to 64 g/L.

With an increase in the ratio of the mass of the powder to the volume of the solution (Figure 5, Table 5), the concentration of nickel ions increased more than twice and reached 146 g/L with a ratio of solid to liquid phases of 3:10 and an acid concentration of 3.0 mol/L, while the use of dilute acid under the same conditions made it possible to obtain a concentration of nickel ions in solution twice as low (67 g/L). The nature of the changes in the concentrations of the main components and the impurities was similar to the change in the concentration of nickel, viz., a threefold increase in the mass of the solid phase. The concentration of metal ions passing into the solution increased by an average of 2.5 times. However, the degree of their extraction decreased to about 75%. It should be noted that the complete dissolution of the grinding waste sample and the transition of all its components into the solution did not occur, even with an increase in the duration of treatment in boiling solution of up to 20 h. The choice of the ratio of solid and liquid phases was determined by the goal to be achieved, i.e., either the most complete extraction of the main components into the solution or obtaining a solution containing a high concentration of the main component.

The size of the grinding waste powder fraction also affected the leaching process. The larger the size of the powder particles, the lower the concentrations of the main elements in the solution for a certain period. Thus, when the 0.063–0.1 mm fraction was dissolved after 5 h of contact, the concentration of nickel ions reached 65 g/L. Acid treatment of the 0.5–1 mm fraction allowed for a lower concentration—only 53 g/L for the same period.

### 2.2. Kinetics of Acid Decomposition of Grinding Waste

The leaching of nickel and other metals into the solution when they interact with sulfuric acid occurs by the following reaction:

Ni + H_2_SO_4_ = NiSO_4_ + H_2_↑

Acid leaching of grinding waste is a heterogeneous process, the rate of which is determined by the rate of diffusion transfer and the rate of chemical reaction of dissolution. When sulfuric acid interacts with metals, hydrogen bubbles are formed, which promote the mixing of the solution near the surface of the solid phase. In addition, given the intensive mechanical mixing of the solution, it can be assumed that the processes of external mass transfer do not affect the speed of the process as a whole. Thus, to assess the limiting stage that determines the rate of leaching of metals from grinding waste, using the example of nickel, kinetic data were processed using equations describing the processes occurring in the diffusion and kinetic regions, and the diffusion region means internal diffusion.

**Figure 6:**
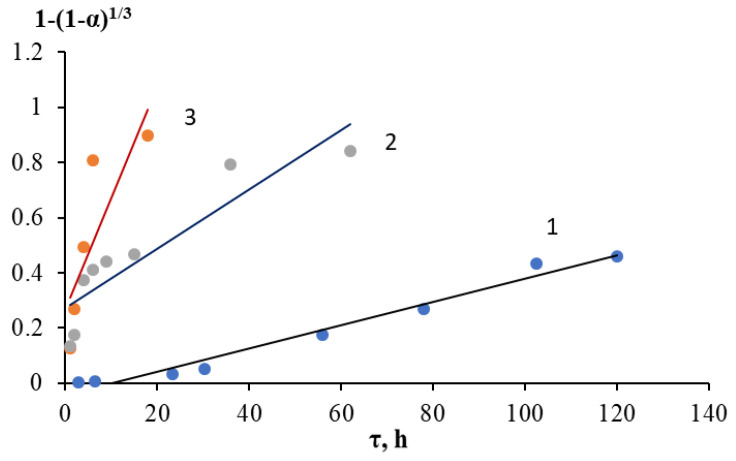
Linearization of kinetic curves of nickel ions leaching into solution in H_2_SO_4_ medium in the coordinates of the Gray–Weddington equation: 1—25 °C; 2—70 °C; 3—100 °C.

**Figure 7:**
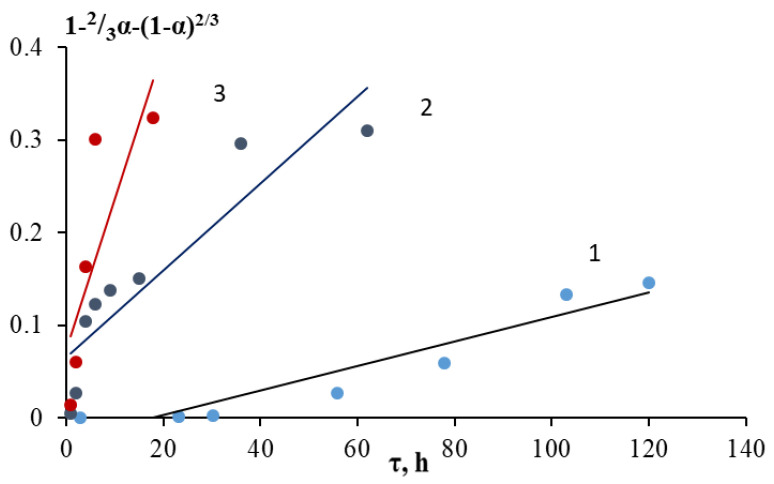
Linearization of kinetic curves of nickel ions leaching into solution in H_2_SO_4_ medium in the coordinates of the Gistling–Brownstein equation: 1—25 °C; 2—70 °C; 3—100 °C.

**Figure 8:**
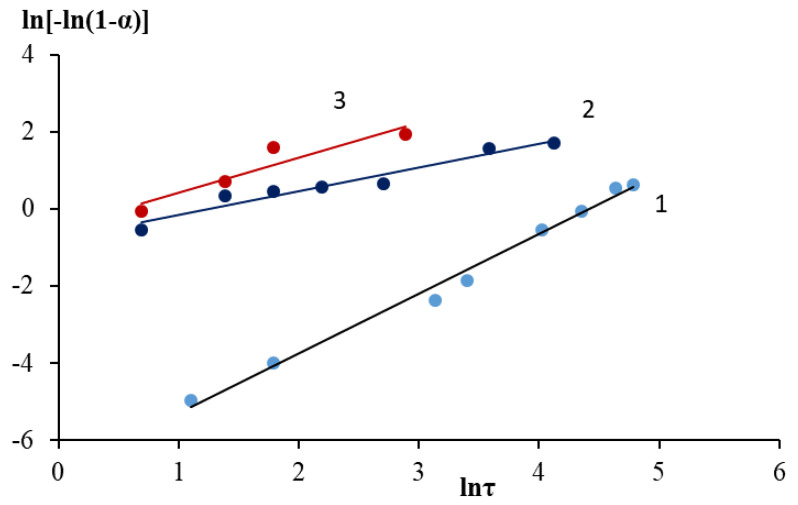
Linearization of kinetic curves of nickel ions leaching into solution in H_2_SO_4_ medium in the coordinates of the Kazeev–Erofeev equation: 1—25 °C; 2—70 °C; 3—100 °C.

The Gray–Weddington equation describes a reaction on the surface of a spherical solid particle, which decreases in size during the reaction, while an insoluble porous layer of the product is formed, which does not affect the diffusion of reagents during the process. The linearization of the kinetic leaching curves of the grinding waste obtained at different temperatures according to the Gray–Weddington equation, as well as the velocity constants, are reflected in Figure 5 and Table 4. In the case of the formation of a dense nonporous layer of the product, the Gistling–Brownstein equation was used (Figure 6, Table 4). Application of the generalized kinetic Kazeev–Erofeev equation (data are presented in Figure 7 and Figure 8 and Table 6) allowed us to identify the limiting stage of the process by evaluating the values of the indicator n in the equation. In a physical sense, this equation describes the transformation probability function both in topochemical reactions and in reactions of a different nature [6].

Judging by the results obtained, the Gray–Weddington and Gistling–Brownstein equations similarly describe the process of leaching grinding waste. The determination coefficients were close except for the data at a temperature of 25 °C. In the first case, the value of R^2^ was slightly higher and was 0.9771, compared with the second case, where R^2^ = 0.9105. Hence, it can be concluded that the process was influenced by both the chemical reaction and the diffusion of reagents through the product layer on the surface of particles (for example, calcium sulfate and rare earth element sulfates) and through a layer of components that are poorly soluble under these conditions. The apparent activation energy calculated by the Arrhenius equation in the temperature range under study using the reaction rate constants of the Gray–Weddington equation was 27.8 kJ/mol. A similar calculation based on the rate constants of the Gistling–Brownstein equation allowed for a close value of 28.6 kJ/mol, which, in both cases, indicated the process in the transition region.

The Kazeev–Erofeev equation most adequately describes the proces, as a result of the approximation of kinetic data. Higher determination coefficients were obtained, which were in the range of 0.9902–0.8772. It should be noted that in all cases, the velocity constant increased significantly with increasing temperature, and the coefficient of determination decreased. The value of the parameter ‘n’ of the Kazeev–Erofeev equation at a temperature of 25 °C was 1.55, that is, more than one, which indicates the predominance of the kinetic component in the speed of the process. With increasing temperature, the value of the coefficient ‘n’ decreased to 0.61 and 0.91 at temperatures of 70 °C and 100 °C, respectively, which indicated that internal diffusion through the product film begins to have a limiting effect on leaching. To remove intra-diffusion inhibition during leaching, the material must be crushed to a fraction less than 0.1 mm in size.

## 3. Experimental

### 3.1. Chemicals Used

The grinding waste was a powder. The mineral acids H_2_SO_4_, HCl, and HNO_3_ were used to dissolve the grinding waste. EDTA, murexide indicator (C_8_H_8_N_6_O_6_), and NH_4_OH solution were used for the titrimetric determination of nickel ions. K_2_Cr_2_O_7_ and Ag_2_SO_4_ were used for the analysis of organic impurities.

### 3.2. Instruments Used

A standard set of metal sieves of 1.0, 0.5, 0.2, 0.1, 0.063, 0.05, and 0.04 mm was used to determine the granulometric composition of the grinding waste. Calcination of the grinding waste was carried out in a muffle furnace (LF-2/13-G1). The morphology and dispersion of powders were studied using a scanning electron microscope (JSM-6510 JEOL). The elemental composition of the grinding waste was determined on the INCA Energy analyzer by Oxford Instruments. A magnetic stirrer (ES-6120) with heating and a reverse refrigerator were used to leach the grinding waste. The metal content in the sample was also determined by an Agilent 5800 spectrometer with the “Multiwave Go Plus” system. The analysis of organic impurities was carried out using an SF-2000 Spectrometer.

### 3.3. Procedure

#### 3.3.1. Sample Preparation

The grinding waste obtained from the industrial company was a powder; no additional grinding was carried out. To remove organic impurities, the initial powder was subjected to heat treatment at 600 °C for 2 h.

#### 3.3.2. Characterization of Solid Waste Samples

The granulometric composition of the powder under study was determined by sieve analysis by sifting the sample through a standard set of sieves and weighing the remaining material on each sieve. A set of sieves with the following diameters of holes was used for sieving: 1.0, 0.5, 0.2, 0.1, 0.063, 0.05, and 0.04 mm. The morphology and dispersion of powders were studied using scanning electron microscopy, and micrographs of the surface of the samples were obtained at an accelerating voltage of 15 kV using a secondary electron detector. Investigation of the elemental composition of the grinding waste was performed by X-ray energy dispersion spectroscopy. The metal content in the sample was also determined by inductively coupled plasma optical emission spectroscopy with microwave sample preparation (the sample was initially dissolved in hydrochloric acid).

#### 3.3.3. Samples Leaching

The dissolution of the grinding waste was carried out in acids (H_2_SO_4_, HCl, HNO_3_, and their mixtures) in the temperature range of 25 to 100 °C with continuous stirring of the solution in a heat-resistant flask with a reverse refrigerator in order to prevent evaporation of the solution. The ratio of grinding powder to solution (P:S) was changed from 1:10 to 3:10. The kinetics of the process of leaching nickel ions into the solution was monitored by the next method: at certain intervals, the liquid phase was selected and analyzed for the content of nickel ions. The determination of organic impurities in solutions after acid treatment of waste due to the presence of lubricating fluids in the grinding waste (chemical oxygen consumption) was carried out by the photometric method.

#### 3.3.4. Method of Metal Ion Determination

The content of all metal ions present in the resulting solution was determined by optical emission spectroscopy of inductively coupled plasma. The content of nickel ions during kinetic investigations was analyzed by the titrimetric method using EDTA.

#### 3.3.5. Description of Kinetic Data

The following equations were used to describe the kinetics of the process of leaching nickel ions into solution and the linearization of experimental data:

Gray–Weddington equation—control of surface chemical reactions [6,30].

1 − (1 − α)^1/3^ = *k*τ (1)

Gistling–Brownstein equation—product layer diffusion control [31,32].

1 − ^2^/_3_α − (1 − α)^2/3^ = *k*τ (2)

Kazeev–Erofeev equation [6].

ln[−ln(1 − α)] = *n*lnτ + ln*k* (3)

where α is the degree of extraction, in fractions of units; *k* is the velocity constant, h^−1^; τ is the time, h; and *n* is the kinetic parameter of the equation.

## 4. Conclusions

As a result of the study of the acid-leaching process of grinding the waste of heat-resistant nickel alloy in metallurgical production, optimal conditions for its opening were selected. Leaching should be carried out in a solution of sulfuric acid with a concentration of 3 mol/L and a temperature of 100 °C for 6 h. In this case, complete extraction of the main component of the nickel alloy into the solution was achieved, the concentration of which reaches 65 g/L when using a fine fraction of 0.063–0.1 mm waste. Preliminary heat treatment of the grinding waste at a temperature of 600 °C reduced the content of organic impurities in it three times, which, in turn, reduced their concentrations in the solution after acid treatment of the powder. As a result of the linearization of the kinetic data of leaching, it was found that the process proceeded in the transition region and was limited by both the reaction rate and intra-diffusion inhibition, as evidenced by the apparent activation energy of 28 kJ/mol. The data obtained are of practical interest to industrial enterprises engaged in the production of metals and the processing of metal-containing man-made waste.

## Figures and Tables

**Table 1:** Granulometric composition of the particles of the initial and heat-treated grinding waste according to the sieve analysis.

**Size, mm**	0.5–1	0.2–0.5	0.1–0.2	0.063–0.1	0.05–0.063	0.04–0.05	<0.04
**Original sample**							
**wt.%**	7.2	35.1	40.2	15.7	1.9	0	0
**Heat-treated sample**							
**wt.%**	8.27	17.98	25.83	12.65	7.90	8.28	19.08

**Table 2:** Elemental composition of the surface of the initial and heat-treated grinding waste powders according to X-ray energy dispersion spectroscopy.

Elements	C	O	Al	Ti	Cr	Co	Ni	Mo	W
**Original samples**									
**wt.%**	14.92	12.50	2.33	2.88	14.57	1.15	36.74	5.13	9.78
**Heat-treated samples**									
**Масс.%**	6.72	9.19	1.36	3.07	15.9	1.12	46.94	8.08	7.62

**Table 3:** Metal content in the grinding waste according to optical emission spectroscopy of inductively coupled plasma.

Elements	Ni	Cr	Co	Mo	Ti	Al	Fe	Re	Ir	V	Ru	Ca	W
**g/kg**	502.0	70.4	68.7	172.3	15.4	71.2	6.8	5.9	1.9	1.4	0.84	0.68	0.42

**Table 4:** The content of metal ions in the solution and leaching efficiency as a result of the leaching of the grinding waste powder in sulfuric acid, depending on temperature.

T (°C)	Parameter	Ni	Cr	Co	Mo	Ti	Al	Fe	Re	Nb	W	Ta	Na
25	C, g/L	45.12	7.52	5.69	1.72	1.62	4.02	0.76	0.01	0.08	1.21	0.05	0.10
	α, %	70	63	75	10	88	56	82	2	-	-	-	-
70	C, g/L	61.63	11.67	7.34	2.41	1.85	5.21	0.93	0.05	0.10	1.59	0.07	0.81
	α, %	96	98	99	14	99.9	73	99.9	8	-	-	-	-
100	C, g/L	64.15	11.88	7.57	2.51	1.85	5.33	0.93	0.05	0.01	1.18	0.03	0.53
	α, %	99.9	99.9	99.9	15	99.9	75	99.9	8	-	-	-	-

**Table 5:** The content of metal ions in the solution as a result of the dissolution of the grinding waste powder in sulfuric acid at a different ratio of the grinding waste and the volume of the solution (P:S).

P:S	Contents of Metal Ions in the Solution (g/L)											
	Ni	Cr	Co	Mo	Ti	Al	Fe	Re	Nb	W	Ta	Na
1:10	64.15	11.88	7.57	2.51	1.84	5.33	0.93	0.05	0.01	1.18	0.03	0.53
2:10	105.46	16.03	13.46	4.14	2.34	9.18	1.45	0.02	0.60	5.22	0.25	0.84
3:10	146.36	23.46	18.97	6.13	4.27	12.88	2.23	0.04	0.90	8.35	0.41	1.17

**Table 6:** Kinetic characteristics of nickel leaching from grinding waste.

T, °C	Gray–Weddington Equation		Gistling–Brownstein Equation		Kazeev–Erofeev Equation		
	*k·*10^3^, h^–1^	*R* ^2^	*k·*10^3^, h^–1^	*R* ^2^	*n*	*k·*10^3^, h^–1^	*R* ^2^
25	4.2	0.9771	1.3	0.9105	1.55	1.1	0.9902
70	10.8	0.8269	4.7	0.8278	0.61	462.0	0.9403
100	40.2	0.6832	16.3	0.6483	0.91	608.6	0.8772

## Data Availability

Not applicable.
